# 1,4-Diazo­niabicyclo­[2.2.2]octane tetra­bromidocuprate(II) monohydrate

**DOI:** 10.1107/S1600536811005289

**Published:** 2011-02-23

**Authors:** Yi Zhang, Bo Wang

**Affiliations:** a Ordered Matter Science Research Center, Southeast University, Nanjing 211189, People’s Republic of China

## Abstract

In the title monohydrated salt, (C_6_H_14_N_2_)[CuBr_4_]·H_2_O, the copper(II) ion is coordinated by the four bromide ions in a flattened tetra­hedral geometry. In the crystal, the cations, anions and water mol­ecules inter­act *via* N—H⋯O, O—H⋯Br and N—H⋯Br hydrogen bonds, forming chains parallel to the *b* axis. The chains are further linked by O—H⋯Br hydrogen bonds into layers parallel to the *bc* plane.

## Related literature

For related structures, see: Wei & Willett (1996 ▶, 2002 ▶); Brammer *et al.* (2002 ▶); Zhang *et al.* (2010 ▶). 
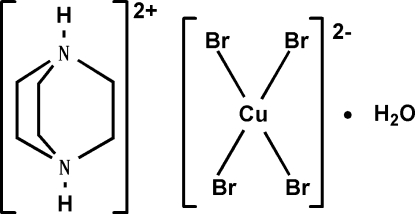


## Experimental

### 

#### Crystal data

(C_6_H_14_N_2_)[CuBr_4_]·H_2_O
*M* *_r_* = 515.39
Monoclinic, 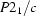

*a* = 9.5171 (19) Å
*b* = 9.5341 (19) Å
*c* = 14.952 (3) Å
β = 93.93 (3)°
*V* = 1353.5 (5) Å^3^
*Z* = 4
Mo *K*α radiation
μ = 13.40 mm^−1^
*T* = 298 K
0.20 × 0.20 × 0.20 mm

#### Data collection

Rigaku SCXmini diffractometer
Absorption correction: multi-scan (*CrystalClear*; Rigaku, 2005 ▶) *T*_min_ = 0.055, *T*_max_ = 0.086
13570 measured reflections
3111 independent reflections
2285 reflections with *I* > 2σ(*I*)
*R*_int_ = 0.124

#### Refinement

*R*[*F* ^2^ > 2σ(*F* ^2^)] = 0.052
*wR*(*F* ^2^) = 0.116
*S* = 1.10
3111 reflections
128 parameters
H-atom parameters constrained
Δρ_max_ = 1.22 e Å^−3^
Δρ_min_ = −1.29 e Å^−3^

### 

Data collection: *CrystalClear* (Rigaku, 2005 ▶); cell refinement: *CrystalClear*; data reduction: *CrystalClear*; program(s) used to solve structure: *SHELXS97* (Sheldrick, 2008 ▶); program(s) used to refine structure: *SHELXL97* (Sheldrick, 2008 ▶); molecular graphics: *SHELXTL* (Sheldrick, 2008 ▶); software used to prepare material for publication: *SHELXTL*.

## Supplementary Material

Crystal structure: contains datablocks I, global. DOI: 10.1107/S1600536811005289/rz2552sup1.cif

Structure factors: contains datablocks I. DOI: 10.1107/S1600536811005289/rz2552Isup2.hkl

Additional supplementary materials:  crystallographic information; 3D view; checkCIF report

## Figures and Tables

**Table 1:** Hydrogen-bond geometry (Å, °)

*D*—H⋯*A*	*D*—H	H⋯*A*	*D*⋯*A*	*D*—H⋯*A*
N2—H2*C*⋯O1*W*	0.91	2.49	3.121 (8)	127
N2—H2*C*⋯O1*W*^i^	0.91	1.98	2.788 (7)	147
O1*W*—H1*WA*⋯Br4^ii^	0.85	2.75	3.456 (5)	141
O1*W*—H1*WA*⋯Br2^ii^	0.85	2.86	3.461 (5)	129
O1*W*—H1*WB*⋯Br1^iii^	0.85	2.55	3.319 (5)	152
N1—H1*C*⋯Br1	0.91	2.61	3.360 (5)	140
N1—H1*C*⋯Br4	0.91	2.92	3.546 (6)	127

Symmetry codes: (i) 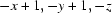; (ii) 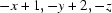; (iii) 
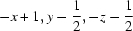
.

**Fig. 1.**
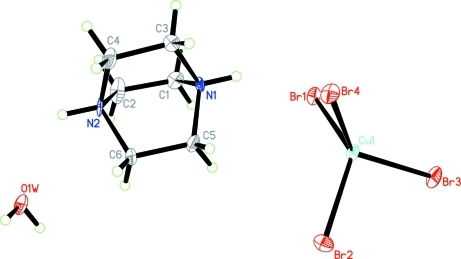
The molecular structure of the title compound, with the atomic numbering scheme. Displacement ellipsoids are drawn at the 30% probability level.

**Fig. 2.**
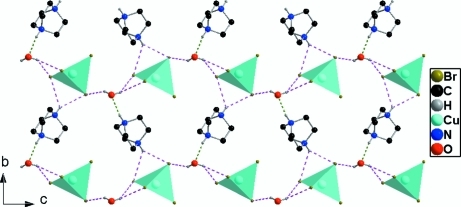
Packing diagram of the title compound viewed along the a axis. H atoms not involved in hydrogen bonding (dashed lines) are omitted.

## supplementary crystallographic information

### Comment

Ferroelectric materials have attracted intensive interest not only due to their versatile technological applications in the field of electronics and optics, but also for their importance to the fundamental scientific research. Recently, monosalts of 1,4-diazabicyclo[2.2.2]octane (dabco) including dabco*HBF_4_*, dabco*HClO_4_* and dabco*HReO_4_* have been reported to have excellent dielectric and ferroelectric properties (Wei & Willett, 1996, 2002; Brammer *et al.*, 2002). Our group has recently reported the compound (dabcoH_2_)_2_Cl_3_[CuCl_3_(H_2_O)_2_].H_2_O (Zhang *et al.*, 2010), which also shows good dielectric and ferroelectric properties. Herein we report the synthesis and crystal structure of the title compound, (dabcoH_2_)CuBr_4_.H_2_O.

The asymmetric unit of the title compound contains one (dabcoH_2_)^2+^ cation, one [CuBr_4_]^2-^ anion and one water molecules (Fig 1). The copper(II) ion has a flattened tetrahedral coordination geometry provided by the four Br^-^ ions, with Cu—Br distances ranging from 2.3598 (12) to 2.4070 (12) Å. Generally, the Cu—Br bond lengths and Br—Cu—Br bond angles in a [CuBr_4_]^2-^ anion are not equal to one another but vary with the environment around the Br atoms. As atoms Br1, Br2 and Br4 are involved in hydrogen bonds, the Cu1—Br3 bond length is significantly shorter than the other Cu—Br bonds. The distortion from the ideal tetrahedral geometry is also indicated by the values of the Br—Cu—Br angles, which range from 96.75 (4) to 133.92 (5)°. The H1C and H2C protons of the 1,4-diazoniabicyclo(2.2.2)octane cation and the H1WA hydrogen atom of the water molecule are engaged in bifurcated N—H···Br, N—H···O and O—H···Br hydrogen bonds (Table 1), forming chains parallel to the *b* axis. The chains are further connected by O—H···Br hydrogen bonds into layers parallel to the (011) plane (Fig. 2).

### Experimental

To a concentrated HBr water solution (50 ml) 1,4-diazabicyclo[2.2.2]octane (10 mmol, 1.12 g) and of CuBr_2_.2H_2_O (10 mmol, 2.60 g) were added with stirring. Brown single crystals of the title compound suitable for X-ray analysis were obtained by slow evaporation of the solvent over a period of a week at room temperature. The dielectric constant of the compound as a function of temperature indicates that the permittivity is basically temperature-independent (*ε*= C/(T–T_0_)), suggesting that the title compound is not ferroelectric or there may be no distinct phase transition occurring within the measured temperature range between 93 K and 362 K (m. p. 99 °C).

### Refinement

All H atoms were fixed geometrically and treated as riding, with C–H = 0.97 Å, N—H = 0.91 Å, O—H = 0.85 Å, and with *U*_iso_(H) = 1.2 *U*_eq_(C, N) or 1.2 *U*_eq_(O).

### Crystal data

**Table:**

(C_6_H_14_N_2_)[CuBr_4_]·H_2_O	*F*(000) = 972
*M**_r_* = 515.39	*D*_x_ = 2.529 Mg m^−^^3^
Monoclinic, *P*2_1_/*c*	Mo *K*α radiation, λ = 0.71073 Å
Hall symbol: -P 2ybc	Cell parameters from 2622 reflections
*a* = 9.5171 (19) Å	θ = 3.0–27.5°
*b* = 9.5341 (19) Å	µ = 13.40 mm^−^^1^
*c* = 14.952 (3) Å	*T* = 298 K
β = 93.93 (3)°	Polyhedron, brown
*V* = 1353.5 (5) Å^3^	0.20 × 0.20 × 0.20 mm
*Z* = 4	

### Data collection

**Table:**

Rigaku SCXmini diffractometer	3111 independent reflections
Radiation source: fine-focus sealed tube	2285 reflections with *I* > 2σ(*I*)
graphite	*R*_int_ = 0.124
Detector resolution: 13.6612 pixels mm^-1^	θ_max_ = 27.5°, θ_min_ = 3.0°
ω scans	*h* = −12→12
Absorption correction: multi-scan (*CrystalClear*; Rigaku, 2005)	*k* = −12→12
*T*_min_ = 0.055, *T*_max_ = 0.086	*l* = −19→19
13570 measured reflections	

### Refinement

**Table:**

Refinement on *F*^2^	Secondary atom site location: difference Fourier map
Least-squares matrix: full	Hydrogen site location: inferred from neighbouring sites
*R*[*F*^2^ > 2σ(*F*^2^)] = 0.052	H-atom parameters constrained
*wR*(*F*^2^) = 0.116	*w* = 1/[σ^2^(*F*_o_^2^) + (0.0225*P*)^2^ + 2.0506*P*] where *P* = (*F*_o_^2^ + 2*F*_c_^2^)/3
*S* = 1.10	(Δ/σ)_max_ < 0.001
3111 reflections	Δρ_max_ = 1.22 e Å^−^^3^
128 parameters	Δρ_min_ = −1.29 e Å^−^^3^
0 restraints	Extinction correction: *SHELXL97* (Sheldrick, 2008), Fc^*^=kFc[1+0.001xFc^2^λ^3^/sin(2θ)]^-1/4^
Primary atom site location: structure-invariant direct methods	Extinction coefficient: 0.0204 (7)

### Special details

**Table:**

Geometry. All e.s.d.'s (except the e.s.d. in the dihedral angle between two l.s. planes) are estimated using the full covariance matrix. The cell e.s.d.'s are taken into account individually in the estimation of e.s.d.'s in distances, angles and torsion angles; correlations between e.s.d.'s in cell parameters are only used when they are defined by crystal symmetry. An approximate (isotropic) treatment of cell e.s.d.'s is used for estimating e.s.d.'s involving l.s. planes.
Refinement. Refinement of *F*^2^ against ALL reflections. The weighted *R*-factor *wR* and goodness of fit *S* are based on *F*^2^, conventional *R*-factors *R* are based on *F*, with *F* set to zero for negative *F*^2^. The threshold expression of *F*^2^ > σ(*F*^2^) is used only for calculating *R*-factors(gt) *etc*. and is not relevant to the choice of reflections for refinement. *R*-factors based on *F*^2^ are statistically about twice as large as those based on *F*, and *R*- factors based on ALL data will be even larger.

### Fractional atomic coordinates and isotropic or equivalent isotropic displacement parameters (Å^2^)

**Table:**

	*x*	*y*	*z*	*U*_iso_*/*U*_eq_	
Br1	0.13288 (8)	1.07551 (7)	−0.32168 (5)	0.0252 (2)	
Br2	0.47455 (8)	1.29945 (9)	−0.17676 (5)	0.0294 (3)	
Br3	0.24026 (10)	1.44329 (8)	−0.35401 (5)	0.0343 (3)	
Br4	0.12020 (9)	1.24314 (8)	−0.10831 (5)	0.0291 (2)	
C1	0.2071 (9)	0.7578 (7)	−0.1967 (5)	0.0250 (18)	
H1A	0.1136	0.7276	−0.2186	0.030*	
H1B	0.2588	0.7837	−0.2479	0.030*	
C2	0.2812 (10)	0.6413 (8)	−0.1464 (5)	0.039 (2)	
H2A	0.2182	0.5619	−0.1423	0.046*	
H2B	0.3620	0.6113	−0.1776	0.046*	
C3	0.1196 (8)	0.8404 (8)	−0.0560 (5)	0.0221 (17)	
H3A	0.0265	0.8058	−0.0751	0.027*	
H3B	0.1094	0.9204	−0.0170	0.027*	
C4	0.2029 (9)	0.7281 (8)	−0.0075 (6)	0.032 (2)	
H4A	0.2322	0.7602	0.0524	0.039*	
H4B	0.1444	0.6456	−0.0023	0.039*	
C5	0.3414 (8)	0.9335 (7)	−0.1063 (5)	0.0265 (19)	
H5A	0.3357	1.0120	−0.0654	0.032*	
H5B	0.3896	0.9644	−0.1579	0.032*	
C6	0.4211 (9)	0.8136 (8)	−0.0601 (6)	0.040 (2)	
H6A	0.5019	0.7893	−0.0932	0.048*	
H6B	0.4551	0.8418	−0.0001	0.048*	
Cu1	0.24156 (9)	1.27018 (9)	−0.24116 (6)	0.0190 (3)	
N1	0.1962 (6)	0.8822 (6)	−0.1354 (4)	0.0164 (13)	
H1C	0.1478	0.9520	−0.1654	0.020*	
N2	0.3277 (6)	0.6913 (6)	−0.0552 (4)	0.0204 (14)	
H2C	0.3753	0.6211	−0.0251	0.024*	
O1W	0.6324 (6)	0.5717 (5)	−0.0241 (3)	0.0316 (14)	
H1WA	0.6670	0.5956	0.0276	0.047*	
H1WB	0.6832	0.6044	−0.0638	0.047*	

### Atomic displacement parameters (Å^2^)

**Table:**

	*U*^11^	*U*^22^	*U*^33^	*U*^12^	*U*^13^	*U*^23^
Br1	0.0381 (5)	0.0188 (4)	0.0181 (5)	−0.0005 (3)	−0.0012 (4)	−0.0011 (3)
Br2	0.0251 (4)	0.0452 (5)	0.0177 (5)	0.0018 (4)	0.0000 (3)	0.0023 (4)
Br3	0.0557 (6)	0.0281 (5)	0.0180 (5)	−0.0065 (4)	−0.0067 (4)	0.0144 (4)
Br4	0.0378 (5)	0.0327 (5)	0.0182 (5)	0.0007 (4)	0.0138 (4)	0.0031 (3)
C1	0.037 (5)	0.028 (4)	0.010 (4)	−0.003 (4)	0.002 (3)	−0.008 (3)
C2	0.077 (7)	0.025 (5)	0.015 (5)	0.015 (5)	0.009 (5)	−0.004 (4)
C3	0.027 (4)	0.029 (4)	0.010 (4)	0.003 (3)	0.003 (3)	−0.004 (3)
C4	0.046 (5)	0.029 (5)	0.024 (5)	0.009 (4)	0.012 (4)	0.010 (4)
C5	0.035 (4)	0.017 (4)	0.026 (5)	−0.007 (3)	−0.007 (4)	0.006 (3)
C6	0.029 (5)	0.035 (5)	0.054 (6)	−0.001 (4)	−0.011 (4)	0.029 (5)
Cu1	0.0288 (5)	0.0193 (5)	0.0087 (5)	−0.0016 (4)	0.0003 (4)	0.0041 (4)
N1	0.020 (3)	0.019 (3)	0.011 (3)	0.005 (3)	−0.002 (2)	0.007 (3)
N2	0.037 (4)	0.011 (3)	0.013 (3)	0.006 (3)	−0.002 (3)	0.008 (3)
O1W	0.045 (3)	0.027 (3)	0.023 (3)	0.000 (3)	0.008 (3)	0.010 (2)

### Geometric parameters (Å, °)

**Table:**

Br1—Cu1	2.4070 (12)	C4—N2	1.469 (10)
Br2—Cu1	2.3726 (13)	C4—H4A	0.9700
Br3—Cu1	2.3598 (12)	C4—H4B	0.9700
Br4—Cu1	2.3792 (13)	C5—N1	1.502 (9)
C1—C2	1.492 (10)	C5—C6	1.512 (10)
C1—N1	1.507 (9)	C5—H5A	0.9700
C1—H1A	0.9700	C5—H5B	0.9700
C1—H1B	0.9700	C6—N2	1.471 (9)
C2—N2	1.483 (9)	C6—H6A	0.9700
C2—H2A	0.9700	C6—H6B	0.9700
C2—H2B	0.9700	N1—H1C	0.9100
C3—N1	1.489 (9)	N2—H2C	0.9100
C3—C4	1.490 (10)	O1W—H1WA	0.8501
C3—H3A	0.9700	O1W—H1WB	0.8500
C3—H3B	0.9700		
			
C2—C1—N1	109.2 (6)	C6—C5—H5B	110.1
C2—C1—H1A	109.8	H5A—C5—H5B	108.4
N1—C1—H1A	109.8	N2—C6—C5	109.6 (6)
C2—C1—H1B	109.8	N2—C6—H6A	109.7
N1—C1—H1B	109.8	C5—C6—H6A	109.7
H1A—C1—H1B	108.3	N2—C6—H6B	109.7
N2—C2—C1	109.0 (6)	C5—C6—H6B	109.7
N2—C2—H2A	109.9	H6A—C6—H6B	108.2
C1—C2—H2A	109.9	Br3—Cu1—Br2	99.61 (5)
N2—C2—H2B	109.9	Br3—Cu1—Br4	133.92 (5)
C1—C2—H2B	109.9	Br2—Cu1—Br4	99.64 (5)
H2A—C2—H2B	108.3	Br3—Cu1—Br1	101.57 (4)
N1—C3—C4	107.9 (6)	Br2—Cu1—Br1	130.64 (5)
N1—C3—H3A	110.1	Br4—Cu1—Br1	96.75 (4)
C4—C3—H3A	110.1	C3—N1—C5	110.3 (5)
N1—C3—H3B	110.1	C3—N1—C1	109.4 (5)
C4—C3—H3B	110.1	C5—N1—C1	109.4 (6)
H3A—C3—H3B	108.4	C3—N1—H1C	109.2
N2—C4—C3	110.9 (6)	C5—N1—H1C	109.2
N2—C4—H4A	109.5	C1—N1—H1C	109.2
C3—C4—H4A	109.5	C4—N2—C6	110.2 (6)
N2—C4—H4B	109.5	C4—N2—C2	108.8 (6)
C3—C4—H4B	109.5	C6—N2—C2	110.7 (6)
H4A—C4—H4B	108.1	C4—N2—H2C	109.0
N1—C5—C6	108.0 (6)	C6—N2—H2C	109.0
N1—C5—H5A	110.1	C2—N2—H2C	109.0
C6—C5—H5A	110.1	H1WA—O1W—H1WB	109.5
N1—C5—H5B	110.1		

### Hydrogen-bond geometry (Å, °)

**Table:**

*D*—H···*A*	*D*—H	H···*A*	*D*···*A*	*D*—H···*A*
N2—H2C···O1W	0.91	2.49	3.121 (8)	127
N2—H2C···O1W^i^	0.91	1.98	2.788 (7)	147
O1W—H1WA···Br4^ii^	0.85	2.75	3.456 (5)	141
O1W—H1WA···Br2^ii^	0.85	2.86	3.461 (5)	129
O1W—H1WB···Br1^iii^	0.85	2.55	3.319 (5)	152
N1—H1C···Br1	0.91	2.61	3.360 (5)	140
N1—H1C···Br4	0.91	2.92	3.546 (6)	127

Symmetry codes: (i) −*x*+1, −*y*+1, −*z*; (ii) −*x*+1, −*y*+2, −*z*; (iii) −*x*+1, *y*−1/2, −*z*−1/2.
